# Effectiveness and prognostic factors of apatinib treatment in patients with recurrent or advanced cervical carcinoma: A retrospective study

**DOI:** 10.1002/cam4.3966

**Published:** 2021-05-13

**Authors:** Hui Yang, Min Chen, Zijie Mei, Conghua Xie, Yunfeng Zhou, Hui Qiu

**Affiliations:** ^1^ Department of Radiation and Medical Oncology Zhongnan Hospital of Wuhan University Wuhan People’s Republic of China; ^2^ Hubei Key Laboratory of Tumor Biological Behaviors Zhongnan Hospital of Wuhan University Wuhan People’s Republic of China; ^3^ Hubei Cancer Clinical Study Center Zhongnan Hospital of Wuhan University Wuhan People’s Republic of China

**Keywords:** angiogenesis, apatinib, cervical carcinoma, efficacy, prognosis, retrospective study

## Abstract

**Background:**

Apatinib is an oral anti‐angiogenic drug, its efficacy and prognosis in cervical carcinoma are unclear. This study evaluates the effectiveness and prognostic factors of apatinib in the treatment of recurrent or advanced cervical carcinoma.

**Methods:**

Patients with recurrent or advanced cervical cancer, who agreed to take apatinib, were recruited into this single‐center and retrospective study, and administrated apatinib with or without combination of chemo‐ or radio‐therapy until progressive disease (PD) or unacceptable toxicity.

**Results:**

From March 2017 to February 2019, 53 patients were reviewed. Among them, 2 (3.77%) patients occurred complete response, 16 (30.19%) patients showed partial response, 27 (50.95%) patients had stable disease, and 8 (15.09%) patients had PD. The objective response rate and disease control rate (DCR) of these patients were 33.96% and 84.91%, respectively. The DCR of patients younger than 50, nonsquamous carcinoma, first‐line apatinib therapy, combined radiotherapy, lesions within radiation field, surgical history, and Eastern Cooperative Oncology Group (ECOG) performance status score of 0 or 1 were significantly higher than other patients (*p* < 0.05). The median progression‐free survival (PFS) and overall survival (OS) were 6.0 months (95% CI: 4.43–7.57) and 8.0 months (95% CI: 6.52–9.48), respectively. The univariable and multivariable analysis showed that the patients with an ECOG performance status score of 2 and further line therapy were associated with poor prognosis in both PFS and OS (PFS: HR =8.35, *p* = 0.000; HR =6.66, *p* = 0.001; OS: HR = 7.40, *p* = 0.000; HR = 3.24, *p* = 0.039), respectively. The most common adverse effects (AEs) were hand‐foot syndrome (35.58%), hypertension (18.87%) and fatigue (15.09%). No grade 3 AEs and drug‐related death occurred.

**Conclusion:**

The efficacy and prognosis of patients who are in good general condition and first‐line apatinib combination therapy may be better than other patients. But further phase III clinical trials should be taken to prove this hypothesis.

## Graphical abstract

The efficacy and prognosis of patients who are generally in good condition and first‐line apatinib combined with radiotherapy may be better than other patients.

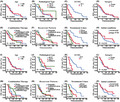

## 1 INTRODUCTION

Cervical cancer is the most common cancer in the female reproductive system.^1^ It can be cured with surgery or radiation at an early stage.^2^ However, when patients occurred recurrent or advanced disease, the average rate of 5‐year overall survival (OS) is approximately 15%.^3^ Due to the lack of effective treatment options, the condition is always incurable.

Tumor‐related neovascularization has been reported related to tumor progression. In cervical cancer, some studies have revealed that tumor neovascularization is linked to poor prognosis. Studies have found that the expression of vascular endothelial growth factor (VEGF) and hypoxia‐inducible factor 1α (HIF‐1α) in high‐grade cervical dysplasia and cervical cancer is increased.^4^, ^5^ Human transforming proteins E6 and E7 are the human papillomavirus oncoproteins. They can promote the growth of tumor neovascularization by enhancing both HIF‐1α and VEGF expression.^6^ The small molecular anti‐angiogenesis drugs have shown efficacy in cervical cancer. As reported by GOG240, compared with women who received chemotherapy alone, women who received bevacizumab therapy in addition to chemotherapy increased the medium OS and progression‐free survival (PFS) by 3.7 and 2.3 months, respectively.^7^ Based on this, the National Comprehensive Cancer Network (NCCN) guidelines recommend bevacizumab combined with chemotherapy to be the first‐line treatment strategy for recurrent and metastatic cervical cancer. However, in China, bevacizumab is only provided at the hospital in big cities, not rural areas. Moreover, it must be administered via intravenous injection every 3 weeks. Furthermore, cervical cancer is more common among underprivileged women in impoverished areas. It seems inconvenient for patients in rural areas to come to the hospital in big cities for bevacizumab treatment every 3 weeks. Thus, there is a need to use other anti‐angiogenic drugs with similar efficacy to bevacizumab but convenient administration.

Apatinib is an oral highly selective tyrosine kinase inhibitor that targets the VEGF receptor‐2, blocks the signal transduction by inhibiting the combination of VEGF and its receptor, and then suppresses tumor angiogenesis.^8^ Apatinib has been approved by the China State Food and Drug Administration (CSFDA) to treat gastric cancer patients who have failed second‐line treatment. Recently, many studies have reported the efficacy of apatinib on various cancer, such as lung cancer,^9^ liver cancer,^10^ breast cancer,^11^ esophageal cancer,^12^ pancreatic cancer,^13^ and ovarian cancer.^14^ In cervical cancer cells, apatinib could inhibit cell proliferation by increasing cell apoptosis and G1 phase arrest. The expression of VEGFR2 in cervical cancer tissues is positively related to the sensitivity of apatinib.^15^ In vivo and in vitro experiments showed apatinib enhanced the sensitivity to paclitaxel.^15^ Based on these biologic rationales, apatinib might affect the treatment of patients with cervical cancer. In the past year, some articles have reported that apatinib may be active in patients with recurrent and metastatic cervical cancer after failure of first‐line treatment.^16^, ^17^, ^18^, ^19^, ^20^, ^21^ However, fewer studies focus on finding which group of patients should be more beneficial from the treatment of apatinib. Herein, the aim of this retrospective study was not only on the efficacy and safety of apatinib in patients with recurrent or advanced cervical cancer but also analysis the various prognostic factors in the different subgroups to find the most beneficial patients.

## 2 MATERIALS AND METHODS

### 2.1 Patient eligibility

This retrospective study enrolled recurrent or advanced cervical cancer patients meeting the inclusion and exclusion criteria in Zhongnan Hospital of Wuhan University from March 2017 to February 2019.

All patients in this study signed informed consent to take apatinib. Meanwhile, they were enrolled based on the following conditions: patients had a pathologically confirmed diagnosis of recurrent or advanced cervical carcinoma; an Eastern Cooperative Oncology Group (ECOG) performance status score of 0, 1, or 2; according to the solid tumor efficacy evaluation standard (RECIST 1.1), patients with at least one measurable lesion; for the patients with recurrent cervical cancer, the disease progression occurred after undergoing radical surgery or radiotherapy, and no bevacizumab used; for the patients with advanced cervical cancer, the disease is International Federation of Gynecology and Obstetrics (FIGO) stage IVB cervical cancer; patients have not received single apatinib or apatinib combined with chemotherapy or radiotherapy. Patients were excluded for the following criteria: patients with uncontrollable high blood pressure, grade II upper coronary heart disease, arrhythmia, and cardiac insufficiency; some factors that lead to the patients cannot persist on oral drugs, such as inability to swallow, nausea and vomiting, chronic diarrhea, and intestinal obstruction; patients with risk of cystorrhagia, gastrointestinal, or vaginal bleeding; patients who have not finished at least one cycle apatinib or evaluated the efficacy.

### 2.2 Treatment and dose modification

For the patients received single apatinib, the dose was initially administered 500 mg once daily; for the patients received apatinib combined with chemotherapy or radiotherapy, the dose was initially administered 500 mg once daily. When the patients cannot tolerate, the dose would be reduced from 500 to 250 mg once daily, or 250 mg every other day. After chemotherapy for four cycles or radiotherapy, the patients were administered apatinib monotherapy till the disease progression or intolerable toxicity.

### 2.3 Efficacy and safety assessments

Progression‐free survival was the primary endpoint. The secondary endpoints were as follows: overall response rate (ORR), disease control rate (DCR), OS, and safety.

Tumor response was evaluated according to RECIST 1.1. It included complete response (CR), partial response (PR), stable disease (SD) and progressive disease (PD). PFS was defined from the time of initiating apatinib treatment to clinical or radiographic progression or death. OS was measured from the time of first apatinib treatment to death or last contact. ORR was measured by the ratio of the number of CR and PR (CR + PR) patients to the total number of patients. DCR was measured by the ratio of the number of CR, PR, and SD (CR + PR + SD) patients to the total number of patients. According to the National Cancer Institute Common Terminology Criteria for Adverse Events, version 5.0 (CTCAE 5.0), safety assessments were recorded from patients’ medical history, laboratory examination results, or telephone follow‐up.

### 2.4 Statistical analysis

Statistical analysis was carried out through SPSS (version 25; SPSS Inc.). Quantitative data are presented as median (95% CI) or the number of patients (percentage). The Wilcoxon or Kruskal–Wallis *H* test was used to analyze the efficacy with different characteristics in two or three groups, respectively. Survival analysis was performed by the Kaplan–Meier method and the log‐rank testing. The Cox regression was used to do the univariate and multivariate analysis. The difference was regarded as statistically significant when *p* < 0.05 (two‐tails).

## 3 RESULTS

### 3.1 The relationship between efficacy and clinicopathological characteristics

A total of 53 patients with recurrent or advanced cervical cancer were included in this retrospective study. On 30 April 2020, 2 patients (3.77%) occurred CR, 16 patients (30.19%) showed PR, 27 patients (50.95%) had SD, 8 patients (15.09%) had PD. The ORR and DCR was 33.96% and 84.91%, respectively. The baseline characteristics in this study were summarized in the second column of Table 1.

**TABLE 1:** The relationship between efficacy and clinicopathological characteristics in this study

Characteristics	No. (%)	CR	PR	SD	PD	*p*‐value	ORR^a^ (%)	DCR^b^ (%)
Total	53	2	16	27	8		33.96	84.91
Age								
≤50	20 (37.74)	1	8	8	3		45.00	85.00
>50	33 (62.26)	1	8	19	5	0.001	27.27	84.85
Pathological type								
Squamous cell carcinoma	41 (77.36)	0	11	22	8		26.83	80.49
Adenocarcinoma	8 (15.09)	1	3	4	0		50.00	100.00
Others	4 (7.55)	1	2	1	0	0.000	75.00	100.00
ECOG performance status score								
0–1	33 (62.26)	2	15	15	1		51.52	96.97
2	20 (37.74)	0	1	12	7	0.000	5.00	65.00
Line of apatinib								
First	21 (39.62)	2	9	10	0		52.38	100.00
Second	18 (33.96	0	5	10	3		27.78	83.33
Further	14 (26.42)	0	2	7	5	0.002	14.29	64.29
Combination therapy								
Combined with chemotherapy	13 (24.53)	1	2	8	2		23.08	84.62
Combined with radiotherapy	12 (22.64)	0	4	8	0		33.33	100.00
None	28 (52.83)	1	10	11	6	0.000	39.29	78.57
Previous radiotherapy of the lesions								
Outside the radiated area	18 (33.96)	0	3	10	5		16.67	72.22
Within the radiated area	9 (16.98)	0	4	5	0		44.44	100.00
Both	26 (49.06)	2	9	12	3	0.011	42.31	88.46
Surgery								
Yes	31 (58.49)	1	11	16	3		38.71	90.32
None	22 (41.51)	1	5	11	5	0.000	27.27	77.27
Initial condition								
Recurrent	43 (81.13%)	1	13	24	5		32.56	88.37
Stage IVB	10 (18.87%)	1	3	3	3	0.000	40.00	70.00

Abbreviations: CR, complete response; DCR, disease control rate; ORR, objective response rate; PD, progressive disease; PR, partial response; SD, stable disease.

^a^ ORR, the ratio of the number of CR and PR (CR + PR) patients to the total number of patients.

^b^ DCR, the ratio of the number of CR, PR, and SD (CR + PR + SD) patients to the total number of patients.

As shown in Table 1, patient efficacy was significantly related to all eight different clinicopathological characteristics. The ORR of patients younger than 50 years old was statistically significant higher with 17.73% increase than that of patients older than 50 years old (*p* = 0.01). The ORR and DCR of patients with squamous carcinoma, adenocarcinoma, and other pathological types were gradually increased (ORR, 26.83% vs. 50.00% vs. 75.00%; DCR, 80.49% vs. 100% vs. 100%) (*p* < 0.001). The patients with an ECOG performance score of 0–1 showed a relatively higher ORR and DCR than patients with a score of 2 (ORR, 51.52% vs. 5.00%; DCR, 96.97% vs. 65.00%; *p* < 0.001). The ORR and DCR of patients using apatinib in first‐line, second‐line, and further‐line therapy were sequentially decreased (ORR, 52.38% vs. 27.78 vs. 14.29%; DCR, 100.00% vs. 83.33% vs. 64.29%; *p* = 0.002). The ORR of the patients treated with apatinib combined with chemotherapy, combined with radiotherapy or apatinib monotherapy were 23.08%, 33.33%, and 39.29%, whereas the DCR of these patients were 84.62%, 100.00%, and 78.57% (*p* < 0.001), respectively. Patients with lesions within the radiated area have significantly higher ORR and DCR than those with lesions outside the radiated area and patients with lesions both within and outside the radiated area, respectively (within radiated area vs. outside radiated area vs. both: ORR: 44.44% vs. 16.67% vs. 42.31%; DCR: 100.00% vs. 72.22% vs. 88.46%; *p* = 0.011). The patients have operation history showed a significantly higher ORR and DCR than those with no operation history (ORR: 38.71% vs. 27.27%; DCR: 90.32% vs. 77.27%; *p* < 0.001). These results indicated that the patients who were younger than 50, nonsquamous carcinoma, ECOG performance status score 0–1, first‐line treatment with apatinib, combination with radiotherapy, and have operation history might benefit more than others.

### 3.2 Survival analysis

**FIGURE 1:**
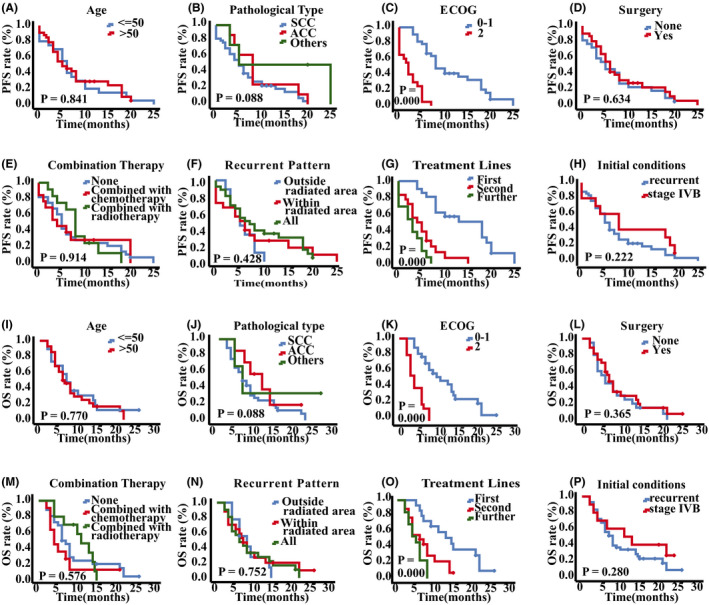
The survival curves of patients in different groups. The survival curves of PFS in different groups of age (A), pathological type (B), ECOG performance status (C), surgery history (D), combination therapy (E), recurrent pattern (F), treatment lines (G), and initial conditions (H), respectively. The survival curves of OS in different groups of age (I), pathological type (J), ECOG performance status (K), surgery history (L), combination therapy (M), recurrent pattern (N), treatment lines (O), and initial conditions (P), respectively. ECOG, Eastern Cooperative Oncology Group; PFS, progression‐free survival

The median PFS and OS in this study were 6.0 (95% CI: 4.43–7.57) months and 8.0 (95% CI: 6.52–9.48) months, respectively. The 1‐year survival rate was 37.0%. As shown in Figure 1 and Table S1, univariate analysis revealed that among the eight clinicopathological features, only the ECOG performance status scores and treatment lines were significantly related to the PFS and OS. The median PFS and OS of patients with an ECOG performance status score of 0–1 were 6 months and 10 months longer than those with a score of 2, respectively (*p* = 0.000). The median PFS and OS of patients using apatinib in first‐line, second‐line, and further‐line therapy were sequentially decreased (PFS: 18.0 months vs. 4.0 months vs. 3.0 months, *p* < 0.001; OS: 21.0 months vs. 6.0 months vs. 5.0 months, *p* < 0.001). The PFS and OS are not statistically significantly different between advanced and recurrent patients (*p* = 0.222 for PFS; *p* = 0.280 for OS).

Since the different combination therapy, pathological type, previous radiotherapy of the lesions, and the operation history affected the efficacy of apatinib, we used multivariate analysis to explore which of them are independent prognostic factor. In the term of PFS, Table 2 showed that the patients with an ECOG performance status score of 2 had a significantly higher cumulative incidence of cancer‐related death than those with a score of 0–1 (HR =8.001, 95% CI: 2.913–21.975; *p* < 0.001). Moreover, when comparing the second or further line therapy with the first‐line treatment, the incidence of death was much higher (second vs. first, HR =3.241, *p* = 0.039; further vs. first, HR =6.660, *p* = 0.001). When comparing combination radiotherapy with no combination therapy, there tended to be different (*p* = 0.056).

**TABLE 2:** Multivariate analysis of PFS and OS

Variables	Multivariate analysis of PFS		Multivariate analysis of OS	
	HR (95% CI)	*p*‐value	HR (95% CI)	*p*‐value
Age				
≤50	1		1	
>50	0.843 (0.428–1.661)	0.622	0.871 (0.429–1.767)	0.702
Pathological type		0.205		0.265
Squamous carcinoma	1		1	
Adenocarcinoma	0.690 (0.252–1.887)	0.469	0.414 (0.123–1.390)	0.154
Others	0.247 (0.050–1.215)	0.085	0.480 (0.091–2.544)	0.389
ECOG performance status score				
0–1	1		1	
2	8.001 (2.913–21.975)	**0.000**	6.914 (2.554–18.722)	**0.000**
Treatment lines		**0.006**		**0.044**
First	1		1	
Second	3.241 (1.063–9.878)	**0.039**	2.629 (0.816–8.476)	0.105
Further	6.660 (2.071–21.418)	**0.001**	4.962 (1.395–17.647)	**0.013**
Combination therapy		0.107		0.064
None	1		1	
Combined with chemotherapy	2.052 (0.794–5.301)	0.138	2.829 (1.040–7.695)	**0.042**
Combined with radiotherapy	2.568 (0.977–6.754)	0.056	2.332 (0.887–6.129)	0.086
Previous radiotherapy of the lesions		0.581		0.871
Within the radiated area	1		1	
Outside the radiated area	0.651 (0.221–1.915)	0.435	0.766 (0.237–2.472)	0.656
Both	0.599 (0.229–1.570)	0.297	0.923 (0.324–2.626)	0.880
Surgery				
None	1		1	
Yes	0.648 (0.298–1.407)	0.273	0.691 (0.307–1.555)	0.371
Initial condition				
Recurrent	1		1	
Stage IVB	1.227 (0.404–3.727)	0.718	1.652 (0.502–5.439)	0.409

Abbreviations: ECOG, Eastern Cooperative Oncology Group; OS, overall survival; PFS, progression‐free survival.

Bold value indicate *p*‐value is statistically significant.

Similarly, in the term of OS, Table 2 showed that the patients with an ECOG performance status score of 2 also had a significantly higher cumulative incidence of cancer‐related death than patients with a sore of 0–1 (HR =6.914, 95% CI: 2.554–18.722; *p* < 0.001). Unlike in the term of PFS, cumulative incidence of cancer‐related death in patients with apatinib as second‐line therapy was significantly higher than those with first‐line therapy (HR = 2.629, 95% CI: 0.816–8.476, *p* = 0.105), while comparing the further line with the first‐line treatment, the incidence of death was much higher (HR = 4.962, 95% CI: 1.395–17.647, *p* = 0.013). Additionally, when comparing apatinib combined with chemotherapy to apatinib monotherapy, the incidence of death was significantly higher (HR = 2.829, 95% CI: 1.040–7.695, *p* = 0.042). These results indicate that the ECOG performance status, treatment line, and combination therapy were independent prognostic factors for patients with recurrent or advanced cervical cancer administrated apatinib.

### 3.3 Adverse effects

Thirty‐three people experienced adverse action, including 19 with the hand‐foot syndrome, 9 with decreased appetite, 10 with hypertension, 8 with fatigue, 2 with hemorrhage, 1 with neutropenia, 2 with a canker sore, and 1 with proteinuria (Table 3). All toxicities were grade 1 to 2. No grade 3 adverse effects and drug‐related death occurred.

**TABLE 3:** Adverse effects (AEs)

AE	Total *n* (%)	No. of patients (%)			
		Grade 1	Grade 2	Grade 3	Grade 4
Hand‐foot syndrome	19 (35.85)	14 (73.68)	5 (26.32)	0	0
Loss of appetite	9 (16.98)	8 (88.89)	1 (11.11)	0	0
Hypertension	10 (18.87)	7 (70.00)	3 (30.00)	0	0
Fatigue	8 (15.09)	7 (87.50)	1 (12.50)	0	0
Hemorrhage	2 (3.77)	2 (100.00)	0	0	0
Neutropenia	1 (1.89)	0	1 (100.00)	0	0
Canker sore	2 (3.77)	2 (100.00)	0	0	0
Proteinuria	1 (1.89)	1 (100.00)	0	0	0
None	20 (37.74)	0	0	0	0

## 4 DISCUSSION

In this study, we demonstrate for the first time that apatinib showed more efficacy in the first‐line treatment of patients with recurrent or advanced cervical cancer than for second‐ or further‐line treatment. Moreover, we identified that ECOG performance status score, combination therapy, and the treatment line were independent prognostic predictor in patients with cervical cancer who were administrated apatinib. The adverse events could be tolerable.

Due to the lack of effective treatments, recurrent or advanced cervical cancer has become a difficulty in treating cervical cancer. Apatinib is an oral anti‐angiogenic drug. Some recent research results show that apatinib has a certain effect on cervical cancer. In 2019, several studies reported the effect of single‐agent apatinib treatment after failure of first‐line treatment in recurrent or advanced cervical cancer.^17^, ^19^, ^20^, ^21^ In 2020, Guo et al^22^ assessed the clinical efficacy and safety of apatinib combined with chemotherapy or concurrent chemo‐brachytherapy as first‐line treatment. In comparison to the results from these studies, ORR, DCR and median PFS, and OS of our study seems a slightly higher than those reported in 2019, but lower than the finding from Guo et al in 2020. The difference in clinical outcomes may be due to that apatinib treatment were used as second‐ or further‐line in the studies in 2019, while used as first‐line in the Guo et al study. In our study, apatinib treatment was used as first‐, second‐ or further‐line. This is also consistent with our finding that apatinib showed more efficacy in the first‐line treatment of patients. Therefore, it is not difficult to conclude that apatinib combined with chemotherapy or radiotherapy as first‐line therapy may be more efficient than monotherapy as second‐line or further‐line therapy.

GOG204 is a phase III clinical study that compared four platinum‐containing dual‐drug combination chemotherapy regimens for the first‐line treatment of relapsed and advanced cervical cancer.^23^ In the GOG204 study, the PFS and OS of the best combination chemotherapy regimen were 5.8 and 12.4 months, respectively.^23^ GOG240 is a phase III clinical study using first‐line chemotherapy combined with bevacizumab versus chemotherapy alone in patients with advanced cervical cancer.^7^ The results showed that the PFS and OS of the bevacizumab combined chemotherapy group were 8.2 and 17 months, respectively.^7^ In our study, the median PFS and OS of patients treated with apatinib as first‐line therapy were 18 and 21 months, respectively, which were higher than the results of GOG204 and GOG240. This suggests that the first‐line treatment of apatinib may be better than the standard first‐line platinum‐containing two‐drug combination chemotherapy, and the first‐line apatinib combined with chemotherapy may be better than bevacizumab combined with chemotherapy.

Our results suggested that the ECOG performance status score and the number of treatment lines were independent risk factors that affect both PFS and OS; in additon, combined chemotherapy was an independent risk factor for OS, but not for PFS. This phenomenon may be related to the limited efficacy of traditional chemotherapy in relapsed or advanced cervical cancer. It can be observed that most patients with combined chemotherapy have poor essential health and low immunity after undergoing multiple chemotherapies. At this time, if more further‐line chemotherapy combined with apatinib was added, there was no doubt that the side effects of this therapy were higher than single drug, and the patient's physical condition and immunity were weakened, so this group of the patients had a poor prognosis. Apatinib can increase the chemotherapy sensitivity of cervical cancer cells to paclitaxel. The first‐line apatinib combined with chemotherapy may result in increasing ORR and DCR, even PFS or OS. The results of Guo et al just corroborated this point.^22^

The PFS and OS in the groups of combined treatment, operation history and previous history of radiotherapy were not statistically significant. Although, the survival analysis was not statistically significant, it can be found from ORR and DCR that apatinib still has better tumor regression in patients with apatinib combined radiotherapy, history of radiotherapy, and the history of surgery. Studies have shown that radiation can cause increased expression of VEGF, activate multiple signal transduction pathways, and promote tumor angiogenesis, leading to resistance to radiotherapy of tumor tissues.^24^, ^25^ Anti‐VEGF monoclonal antibody can counteract the radiotherapy resistance caused by the increase of the VEGF level.^26^ Therefore, radiotherapy combined with antiangiogenic drugs may increase the radiotherapy sensitivity of the tumor. However, our results found that combined radiotherapy with apatinib did not show a long time tumor controlled. It might be because, after radiotherapy combined with apatinib, the time for apatinib monotherapy to control tumor growth was not enough. After radiotherapy, it might be necessary to combine other drugs with apatinib to improve tumor control time and OS.

In this study, the most common side effects were hand‐foot syndrome, hypertension, and fatigue. This result was consistent with the common side effects of apatinib reported in other studies.^20^ The RTOG0417 was a phase II clinical study, which used bevacizumab combined with radiotherapy and cisplatin concurrent chemotherapy as first‐line therapy in locally advanced cervical cancer.^27^ In RTOG0417 study reported that the most common side effect was bone marrow suppression, in which the proportion of grade 3 bone marrow is 22.45%, the incidence of grade 4 bone marrow suppression was 6.12%.^28^ Regarding the combination of bevacizumab and chemotherapy, the GOG240 study has found that in the group of bevacizumab combined with chemotherapy, the incidence of the fistula was 15% and only 1% in the group of chemotherapy alone.^7^ Although there were no incidents requiring emergency surgery due to fistula, the incidence of grade 3 fistula in the group of bevacizumab combined chemotherapy was 6%, much higher than that in the chemotherapy alone group (0.45%).^7^ In this study, apatinib monotherapy or combination therapy did not show grade 3–4 adverse reactions. This indicates that the adverse reactions of apatinib combined treatment may be lighter than bevacizumab and need further extensive sample studies to confirm.

In conclusion, apatinib was an effective drug in patients with recurrence or advanced cervical cancer regardless of a single drug or combined radiotherapy or chemotherapy, and it was well tolerated. After first‐line use of apatinib combined with radiotherapy, taking apatinib in combination with another drug as a maintenance treatment may effectively improve the ORR, DCR, PFS, and OS. However, further studies in Phase III clinical trials are still needed to determine the specific combination of apatinib in cervical cancer.

## CONFLICT OF INTEREST

The authors declare no competing financial interest.

## AUTHOR CONTRIBUTION

Hui Yang, Hui Qiu, Conghua Xie, and Yunfeng Zhou conceived the study. Hui Yang and Min Chen analyzed the patient data. Hui Yang and Zijie Mei wrote the manuscript. Qiuhui, Conghua Xie, and Yunfeng Zhou completed the final review of the manuscript. All authors contributed to discussing and approving the manuscript.

## ETHICS APPROVAL AND CONSENT TO PARTICIPATE

The study was approved by the Ethics Committee of Zhongnan Hospital, Wuhan University, Hubei, China. All patients included in the study signed informed consent to take apatinib. Verbal informed consent was obtained from all patients for data collection.

## Supporting information

Table S1

## Data Availability

According to patient privacy protection and ethical restrictions, the data will not be disclosed. However, if there is need, it can be obtained from the corresponding author.
