# Supplementary material for: Effectiveness and prognostic factors of apatinib treatment in patients with recurrent or advanced cervical carcinoma: A retrospective study
Source: Cancer Med. 2021 May 13;10(13):4282–90. doi: 10.1002/cam4.3966 (PMC8267132; doi:10.1002/cam4.3966)
Supplement: Supplementary file 1 — Table S1 [file CAM4-10-4282-s001.doc]

**Supplementary Table S1** Univariable analysis of PFS and OS

| **Variable** | **No.(%)** | **PFS (median, 95% CI)** | **P-value** | **OS (median, 95% CI)** | **P-value** |
| --- | --- | --- | --- | --- | --- |
| **Total patients** |  | 6.0(4.43-7.57) |  | 8.0(6.52-9.48) |  |
| **Age** |  |  |  |  |  |
| <=50 | 20 | 6.0(4.57-7.43) |  | 7.0(4.81-9.19) |  |
| >50 | 33 | 5.0(2.21-7.79) | 0.841 | 8.0(5.29-10.71) | 0.770 |
| **Pathological type** |  |  |  |  |  |
| Squamous carcinoma | 41 | 5.0(3.21-6.79) |  | 7.0(5.16-8.84) |  |
| Adenocarcinoma | 8 | 8.0(5.60-10.40) |  | 11.0(5.15-16.85) |  |
| Others | 4 | 5.0(0.00-19.37) | 0.088 |  | 0.247 |
| **ECOG performance status score** |  |  |  |  |  |
| 0-1 | 33 | 8.0(5.67-10.33) |  | 14.0(11.48-16.52) |  |
| 2 | 20 | 2.0(1.28-2.72) | **0.000** | 4.0(2.57-5.43) | **0.000** |
| **Treatment lines** |  |  |  |  |  |
| First | 21 | 18.0(11.08-24.92) |  | 21.0(15.45-26.55) |  |
| Second | 18 | 4.0(1.23-6.78) |  | 6.0(0.00-12.24) |  |
| Further | 14 | 3.0(0.42-5.58) | **0.000** | 5.0(3.24-6.76) | **0.000** |
| **Combination therapy** |  |  |  |  |  |
| None | 28 | 5.0(3.97-6.03) |  | 7.0(5.44-8.56) |  |
| Combined with chemotherapy | 13 | 4.0(0.04-7.96) |  | 5.0(1.48-8.52) |  |
| Combined with radiotherapy | 12 | 8.0(6.40-9.60) | 0.914 | 13.0(8.29-17.71) | 0.576 |
| **Previous radiotherapy of the lesions** |  |  |  |  |  |
| Within the radiated area | 9 | 5.0(2.08-7.92) |  | 8.0(2.16-13.84) |  |
| Outside the radiated area | 18 | 5.0(2.23-7.77) |  | 7.0(5.08-8.92) |  |
| Both | 26 | 6.0(3.15-8.86) | 0.428 | 8.0(1.91-14.09) | 0.752 |
| **Surgery** |  |  |  |  |  |
| None | 22 | 5.0(0.42-9.58) |  | 6.0(2.55-9.45) |  |
| Yes | 31 | 6.0(4.46-7.54) | 0.634 | 8.0(6.42-9.59) | 0.365 |
| **Initial condition** |  |  |  |  |  |
| Recurrent | 43 | 5.0(3.57-6.43) |  | 8.0(6.25-9.75) |  |
| Stage IVB | 10 | 8.0(1.93-14.07) | 0.222 | 11.0(0.93-21.07) | 0.280 |
